# Arctigenin prevents monocrotaline-induced pulmonary arterial hypertension in rats

**DOI:** 10.1039/c8ra07892k

**Published:** 2019-01-02

**Authors:** Wei-Long Jiang, Xiao Han, Yu-Feng Zhang, Qing-Qing Xia, Jia-Ming Zhang, Feng Wang

**Affiliations:** Department of Respiration, Jiangyin Hospital of Traditional Chinese Medicine, Jiangyin Hospital Affiliated to Nanjing University of Chinese Medicine Wuxi City Jiangsu Province 214400 China jwljytcm@163.com zyfjytcm@163.com xqqjytcm@163.com; Department of Cardiology, Jiading District Central Hospital Affiliated to Shanghai University of Medicine and Health Sciences Shanghai 201800 China hxjdqzxyy@163.com; Department of Emergency, Wuxi People's Hospital Affiliated to Nanjing Medical University Wuxi City Jiangsu Province 214023 China jiamingsd123@163.com (+86) 510-82700775 (+86) 510-82700775; Department of Neurology, Shanghai General Hospital Affiliated to Shanghai Jiaotong University Shanghai 200080 China wfwangfeng@yahoo.com (+86) 21-63240090 (+86) 21-63240090

## Abstract

The hallmark features of the development of pulmonary arterial hypertension (PAH) include the proliferation of pulmonary vascular smooth muscle cells, oxidative stress, inflammation, and pulmonary artery remodeling. Arctigenin is a bioactive component of *Arctium lappa* that exerts anti-inflammatory and antiproliferative effects in several diseases; however, its effects on pulmonary arteries are still unclear. This study aimed to investigate the efficacy of arctigenin to prevent PAH. Rats injected with monocrotaline (MCT) progressively developed PAH. Arctigenin treatment (50 mg per kg per day, intra-peritoneally) ameliorated right ventricular systolic pressure and pulmonary arterial remodeling, decreased the expression of inflammatory cytokines, and limited the proliferation of pulmonary vascular smooth muscle cells in lungs. Mechanistically, arctigenin effectively inhibited the MCT-induced elevation of NLRP3, caspase-1, and interleukin 1-beta expression in the lungs. These results indicate that arctigenin ameliorates MCT-induced PAH, at least in part, through exerting its anti-inflammatory, antioxidant, and antiproliferative effects, which inhibit the NLRP3 inflammasome signal pathway in rats.

## Abstract 2

Arctigenin ameliorates monocrotaline-induced pulmonary arterial hypertension, at least in part, through exerting its anti-inflammatory, antioxidant, and antiproliferative effects, which inhibit the NLRP3 inflammasome signal pathway in rats.

## Graphical abstract

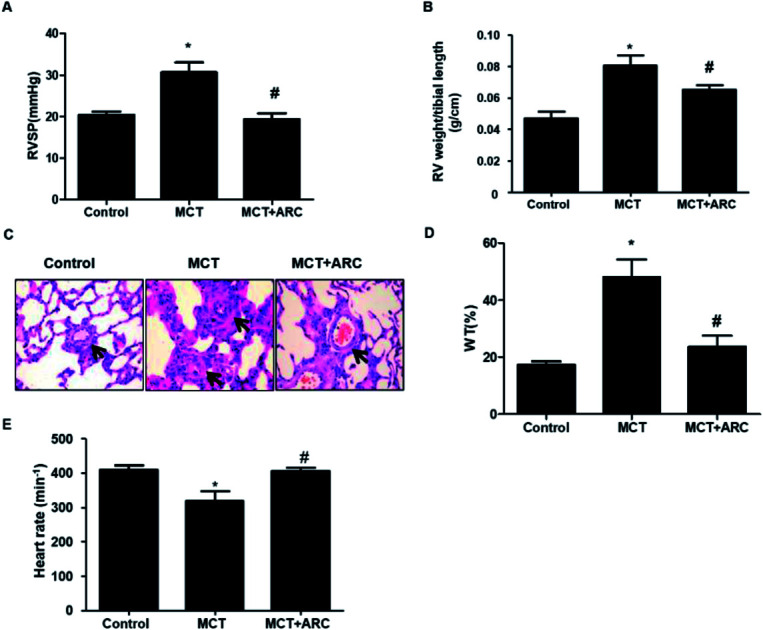

## Introduction

Pulmonary arterial hypertension (PAH) is a progressive and fatal disease featuring structural changes in the pulmonary vasculature, which may induce a severe increase in pulmonary arterial pressure, right ventricular (RV) failure, and death.^1^ Although the pathogenesis of PAH is still unclear, evidence suggests that PAH development is associated with inflammatory activation, endothelial dysfunction, and vascular remodeling, including cellular proliferation in both the intima and media.^2^ Current therapies for chronic PAH, such as endothelin receptor antagonists and phosphodiesterase inhibitors, are mainly designed to reduce pulmonary arterial resistance by inducing vasodilation. However, these approaches mainly induce symptomatic relief and do not have a satisfactory clinical outcome.^3^

The proinflammatory cytokine interleukin 1-beta (IL-1β) has a vital role in PAH.^4,5^ The NLRP3 inflammasome, consisting of the NLR protein NLRP3, an adapter ASC, and pro-caspase-1, has been implicated in the activation of IL-1β and PAH.^6^ Arctigenin (ARC) is a bioactive component of *Arctium lappa*, which has various biological properties including anticancer activity, antioxidant activity, and anti-inflammatory activity.^7^ Our previous study reported that ARC may exert neuroprotective effects in experimental stroke through inhibiting neuroinflammation.^8^ Moreover, a recent study demonstrated that ARC attenuated lipopolysaccharide (LPS)-induced acute lung injury in rats by inhibiting oxidative stress and inflammation.^9^ Most recently, ARC was reported to attenuate stroke by inhibition of the NLRP3 inflammasome.^10^ Above all, pharmacokinetic study of arctigenin in rat has shown that optimal dose of ARC at 50 mg kg^−1^ may deliver delivers effective levels of the compound to the rats.^11^ Given these findings, the present study was designed to investigate whether ARC attenuates PAH in rats by inhibiting the NLRP3 inflammasome. Rats develop severe PAH after a single injection of monocrotaline (MCT), which mimics several key aspects of the primary and secondary forms of human pulmonary hypertension, including vascular remodeling, proliferation of pulmonary artery smooth muscle cells (PASMCs), oxidative stress, and upregulation of inflammatory cytokines. We investigated whether ARC exerts beneficial effects on PASMC proliferation and inflammation.

## Materials and methods

### Preparation of arctigenin

Arctigenin was purchased from Sigma-Aldrich (St. Louis, MO, USA) and the chemical structure is as follows:
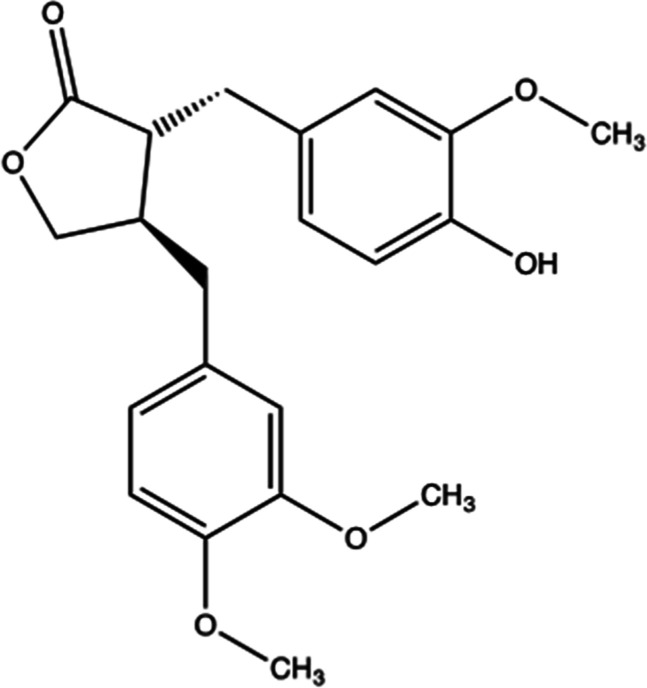


### Animal model

Animal use protocols were approved by the Nanjing University of Chinese Medicine Animal Care and Use Committee. Male Sprague-Dawley rats (200–250 g) were provided by the Nanjing University of Chinese Medicine Laboratory Animal Center (Nanjing, China). This research protocol was approved by the Institutional Animal Care Committee at Jiangyin Hospital of Traditional Chinese Medicine (201534).

Adult rats were randomly assigned to the following groups (*n* = 6 animals in each group) to receive either vehicle or ARC: (1) control animals (Control); (2) MCT (60 mg kg^−1^ SC, once)-treated animals (MCT); or (3) MCT-treated animals receiving ARC (50 mg per kg per day, ip) after MCT treatment (ARC). For the MCT group, MCT was given once 24 h before ARC treatment and saline was administered instead of ARC in the same manner for 28 days. The dose of ARC was based on previous studies, in which 50 mg kg^−1^ of arctigenin pretreatment significantly attenuated LPS-induced acute lung injury in mice.^9^

### Assessment of hemodynamics

Four weeks after MCT injection, rats were anesthetized with pentobarbital (60 mg kg^−1^, ip). Right ventricle systolic pressure (RVSP) was evaluated by right heart puncture with a microtip Millar pressure catheter.^12^ Heart rate was measured by an echocardiography machine (VisualSonics, Canada). After hemodynamic analysis, pieces of the left lung and isolated segments of the small intrapulmonary arteries were removed for histological evaluation or biochemical measurements.

### Measurement of RV hypertrophy

After dissection of the heart, the RV wall was weighed after separation from the left ventricular (LV) wall and the ventricular septum. The ratio of the RV free wall weight divided by the length of the tibia was calculated as an index of RV hypertrophy (which is unaffected by changes in body weight or LV mass). Lung tissues were fixed in 4% paraformaldehyde, embedded in paraffin, and sectioned. The sections were examined by light microscopy (Inverted Fluorescence Microscope, Olympus, Japan) after hematoxylin and eosin (HE) staining. Pulmonary arteries with an external diameter of 25–100 μm were selected for morphometric analysis.^12^ Medial wall thickness was calculated as follows: medial thickness (%) = medial wall thickness/external diameter × 100. Twenty randomly selected vessels from each rat were evaluated, and the mean value was calculated.

### Cell proliferation and apoptosis assays

Immunofluorescent labeling for α-smooth muscle actin was performed to visualize PASMCs. Cell proliferation was also assessed in the walls of distal pulmonary vessels by use of a monoclonal antibody against proliferating cell nuclear antigen (PCNA). Distal pulmonary vessels were identified by morphology. The percentage of PCNA-positive cells was calculated in 10 randomly chosen fields. To assess the antiproliferative effects of ARC *in vitro*, primary human PASMCs were stimulated *in vitro* with platelet derived growth factor (PDGF; 10 ng mL^−1^) in the presence or absence of ARC (1 μg mL^−1^). The inhibitory effect of ARC on cell proliferation was tested with a CCK-8 kit. In brief, PASMCs were seeded on 96-well plates at 80% confluence and treated with PDGF with or without ARC. Viable cells were detected by CCK-8, where the absorbance was assessed at 450 nm with a microplate reader (TECAN, Salzburg, Austria). In addition, the effects of ARC on cell apoptosis of PASMCs were investigated by TUNEL and quantified by flow cytometry.

### Measurement of oxidative stress levels

Lung tissues were collected and homogenized in ice-cold RIPA lysis buffer with a Polytron homogenizer. The homogenates were centrifuged at 3000 rpm at 4 °C for 15 min. The supernatant was collected for superoxide dismutase (SOD) and malondialdehyde (MDA) measurements. SOD activity was measured with SOD assay kits (Jiancheng Bio-Technology, Nanjing, China) and was expressed as U mg^−1^ protein. The lipid peroxide product MDA, a marker for lipid peroxidation and a stable indicator of oxidative stress, was examined by thiobarbituric acid (TBA) assay using an MDA kit (Jiancheng Bio-Technology, Nanjing, China) and expressed as nmol mg^−1^ protein.

### Western blotting

Lung tissues were collected and homogenized as described previously and then centrifuged at 10 000×*g* for 20 min. Equal amounts of total protein mixed with loading buffer were separated by 10% sodium dodecyl sulphate-polyacrylamide gel electrophoresis (SDS-PAGE) and electrophoretically transferred to polyvinylidene difluoride (PVDF) membranes (Millipore, Billerica, MA, USA). The membranes were then blocked with 5% nonfat milk for 1 h and incubated with primary antibodies against β-actin (1 : 1000, Cell Signaling Technologies, Beverly, MA, USA); caspase-1 (1 : 500, Cell Signaling Technologies), NLRP3 (1 : 500, Cell Signaling Technologies) and IL-1β (1 : 1000, Cell Signaling Technologies). Furthermore, the membranes were incubated with IDye800CW conjugated secondary antibody. Protein bands were captured by Odyssey imaging system (LICOR).

### Quantitative real-time PCR

Quantitative real-time PCR (qRT-PCR) was used to analyze mRNA expression, as reported previously. The primers were as follows:

IL-1β (sense 5′-CACCTCTCAAGCAGAGCACAG-3′; antisense 5′-GGGTTCCATGGTGAAGTCAAC-3′); IL-6 (sense 5′-AGCCACTGCCTTCCCTACTTCA-3′; antisense 5′-GCCATTGCACAACTCTTTTCTCA-3′); MCP-1 (sense 5′-CTCTTCCTCCACCACTATGC-3′; antisense 5′-CTCTGTCATACTGGTCACTTC-3′); ICAM-1 (sense 5′-CCTTCCTCACCGTGTACTGG-3′; antisense 5′-AGCGTAGGGTAAGGTTCTTGC-3′); and β-actin (sense 5′-GGAGATTACTGCCCTGGCTCCTA-3′; antisense 5′-GACTCATCGTACTCCTGCTTGCTG-3′). Relative expression was normalized with β-actin.

### Statistical analysis

Data were normalized to the respective control mean values and expressed as means ± SEM. Statistical analyses of data were performed by analysis of variance followed by the Scheffe's post hoc test for multiple comparisons. A value of *P* < 0.05 was considered statistically significant.

## Results

### ARC prevents the MCT-induced increase in RVSP, RV hypertrophy, and pulmonary artery remodeling

**Fig. 1:**
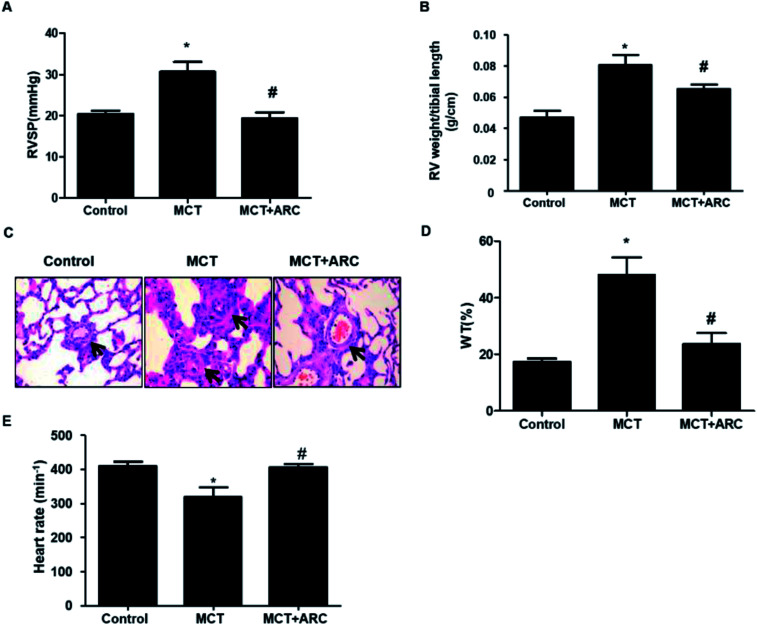
Effects of ARC on MCT-induced pulmonary hypertension. (A) Pulmonary hypertension developed progressively in MCT-injected rats, as shown by increased RV systolic pressure at 21 days post-MCT injection. ARC treatment prevented the development of pulmonary hypertension. (B) RV hypertrophy was indicated by the RV weight : tibia length ratio in rats. ARC treatment prevented RV hypertrophy. (C) Representative hematoxylin and eosin (HE) staining images comparing the arteriole wall thickness in rats. (D) Quantification analysis of percent medial thickness for arterioles (25–100 μm in external diameter) show that ARC reduced the medial wall thickness in MCT-treated rats compared with Controls. (E) MCT-treated rats showed a reduced heart rate compared with Controls; however, no differences were found between untreated MCT and ARC-treated counterparts. Data are expressed as the mean ± SEM. *P < 0.05 *vs.* Control; #*P* < 0.05 *vs.* no ARC.

MCT-exposed rats developed significant pulmonary hypertension 28 days after MCT injection. ARC treatment significantly prevented RVSP in MCT-injected rats over a 4 week period (Fig. 1A). In the MCT groups, significant RV hypertrophy and increased medial thickness of pulmonary arteries developed as a consequence of PAH. These pathological changes were attenuated by ARC treatment (Fig. 1B–D). MCT-treated rats showed a reduced heart rate compared with Controls; however, no differences were found between untreated MCT and ARC-treated counterparts (Fig. 1E).

### ARC inhibits the proliferation of PASMCs

**Fig. 2:**
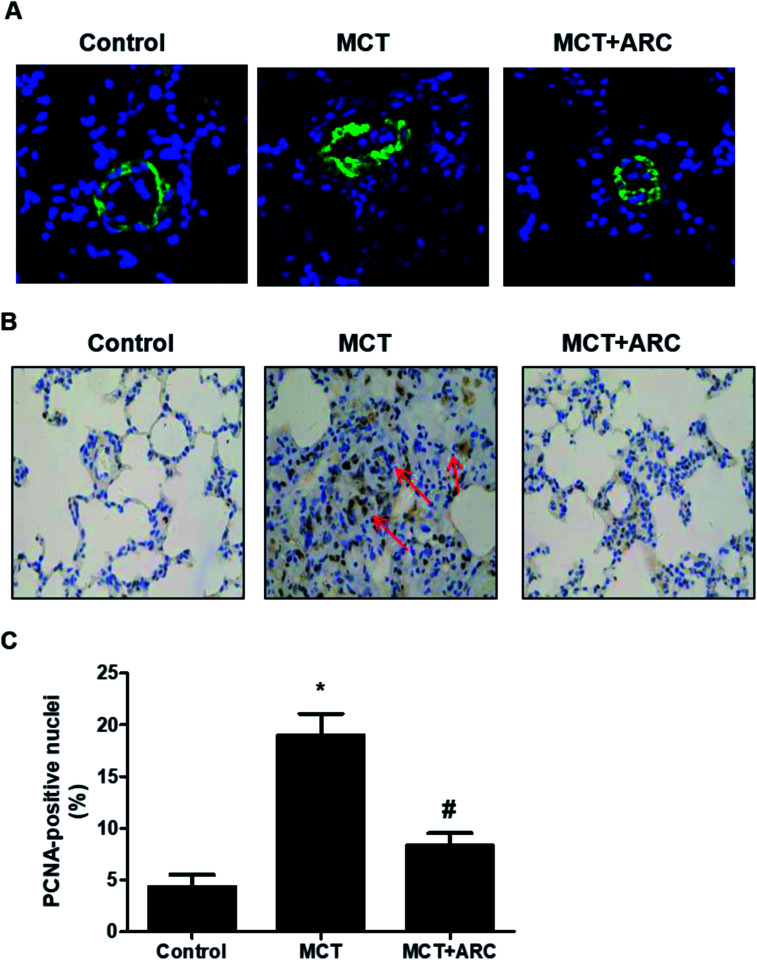
ARC inhibits the proliferation of PASMCs in MCT-induced pulmonary hypertension in rats. (A) Immunofluorescent labeling (green) for α-smooth muscle actin was used to identify vascular smooth muscle cells. (B) PCNA-positive nuclei (brown) in the wall of small pulmonary arteries of MCT- and MCT plus ARC-treated rats. (C) Quantification analysis for (B). Data are expressed as the mean ± SEM. *P < 0.05 *vs.* Control; #*P* < 0.05 *vs.* no ARC.

**Fig. 3:**
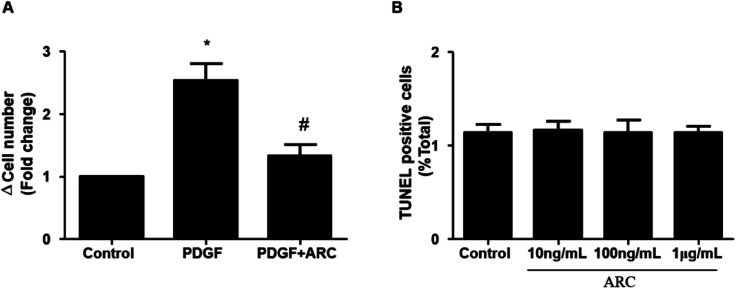
ARC inhibits the proliferation of PASMCs *in vitro*. (A) PDGF (10 ng mL^−1^ for 48 h) stimulates human PASMC proliferation, which is prevented by ARC (10 mol L^−1^). Data are the mean ± SEM. *P < 0.05 *vs.* no ARC; #*P* < 0.05 *vs.* PDGF only. (B) ARC does not stimulate apoptotic cell death in PASMCs.

In the MCT groups, a markedly increased smooth muscle cell mass was observed in the small pulmonary arteries at 28 days after MCT injection. ARC treatment prevented the increase in PASMC mass in the vessels of MCT-treated rats (Fig. 2A). Medial hypertrophy of pulmonary resistance vessels occurred in parallel to an increased number of PCNA-positive proliferating vascular cells in MCT-induced pulmonary hypertension. However, in line with normalization of the PASMC mass, the number of PCNA-positive cells was significantly reduced in ARC-treated animals (Fig. 2B and C). *In vitro* experiments indicated that ARC prevented PDGF (10 ng mL^−1^ for 48 h) and stimulated the proliferation of cultured PASMCs (Fig. 3A). At the concentration range in which the antiproliferative effects were evident, ARC did not induce apoptosis in PASMCs (Fig. 3B).

### ARC attenuates oxidative stress in the lungs

**Fig. 4:**
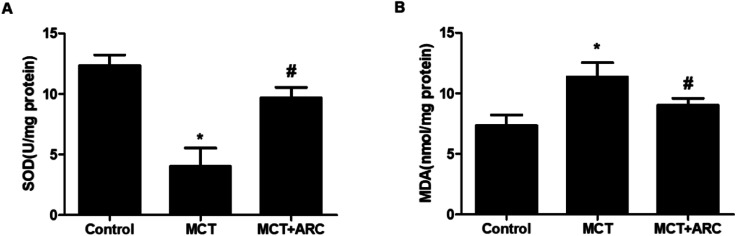
ARC attenuates oxidative stress in rat lungs. ARC suppresses oxidative stress in the lungs of MCT-treated rats. (A) SOD activity in the MCT group was lower than in the Control group, but in the ARC group, it was higher than in the MCT group. (B) The MDA level in the lungs of the MCT group was higher than in the Control group, and in the ARC group, it was lower than in the MCT group. Data represent the mean ± SEM. *P < 0.05 *versus* Control group; #*P* < 0.05 *versus* MCT group. *n* = 6 rats per group.

Ample evidence suggests that oxidative stress has a causal role in PAH.^13^ The inhibition of oxidative stress by ARC (50 mg per kg per day) was associated with the preservation of SOD activity, as well as the downregulation of MDA. SOD activity was inhibited by MCT, and ARC treatment reversed this inhibition (Fig. 4A). Furthermore, as shown in Fig. 4B, MCT induced an elevation of lung MDA levels, whereas ARC inhibited this elevation.

### ARC attenuates inflammatory gene expression

**Fig. 5:**
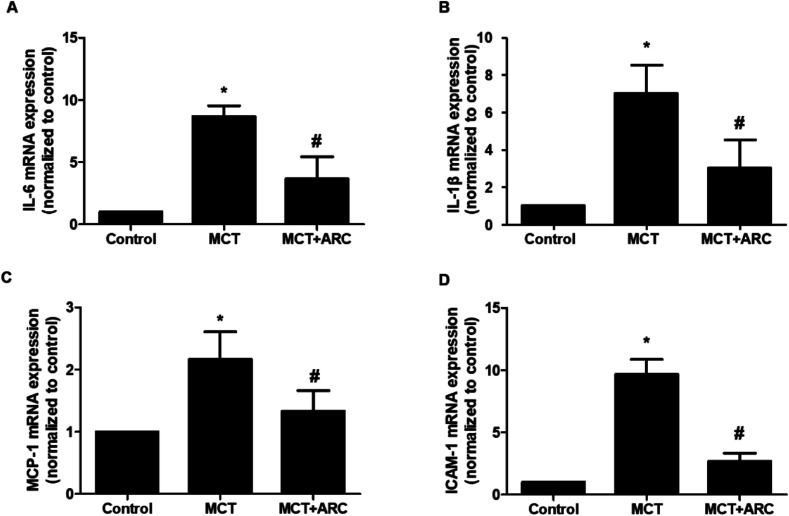
Analysis of mRNA expressions of inflammatory factors in the lungs of Control rats and MCT-injected rats (28 days post-MCT). Analysis of mRNA expressions was performed by real-time quantitative RT-PCR. GAPDH was used for normalization. The mRNA expression of (A) IL-6, (B) IL-1β, (C) MCP-1, and (D) ICAM-1. Data are shown as the mean ± SEM. *P < 0.05 *vs.* Control; #*P* < 0.05 *vs.* no resveratrol (*n* = 6 for each group).

The mRNA expressions of IL-1β, IL-6, MCP-1, and ICAM-1 in the lungs of MCT-treated rats were upregulated in MCT-induced PAH rats, whereas ARC treatment significantly attenuated the expression of each inflammatory marker (Fig. 5A–D).

### ARC inhibits inflammasome activation in MCT-induced PAH rats

**Fig. 6:**
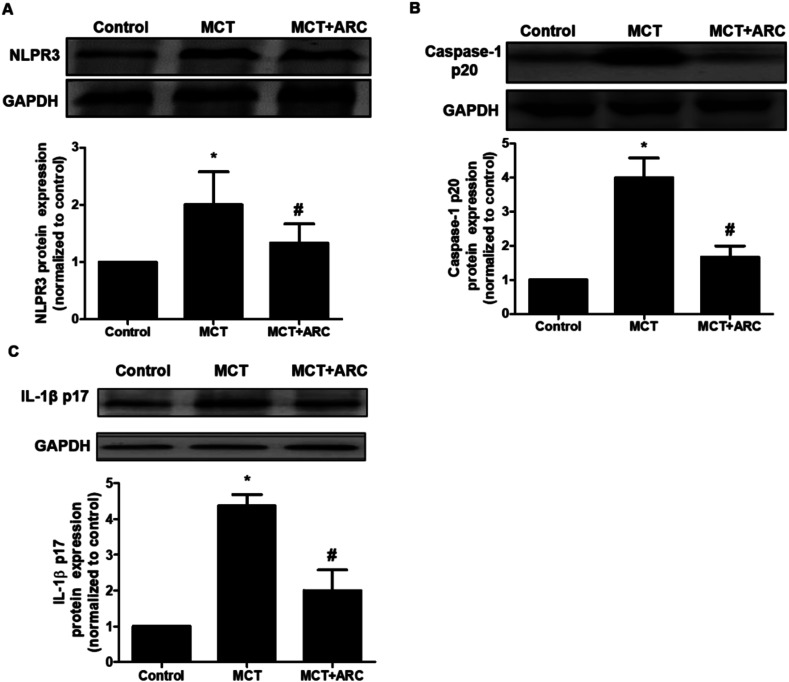
ARC inhibits lung NALP3 inflammasome activation in MCT-treated rats. Western blot showing lung NALP3, caspase-1 and IL-1β expression 4 weeks after MCT injection in different groups of rats. Relative protein levels of NALP3 (A), caspase-1 (B) and IL-1β (C) were determined after normalization with GAPDH. Data are the mean ± SEM. *P < 0.05 *versus* Control group; #*P* < 0.05 *versus* MCT group. *n* = 6 per group.

It was reported that NLRP3 inflammasome activation was tightly associated with PAH. As shown in Fig. 6, compared with the Control group, the MCT group had significantly increased NLRP3, caspase-1 p20 (active subunit), and IL-1β protein levels, whereas ARC treatment reduced these protein levels in the lungs. These findings indicated that ARC may reduce inflammation in rats with MCT-induced PAH partly by inhibiting NLRP3 inflammasome activation.

## Discussion

ARC, an extract from *Arctium lappa* L., has a variety of pharmacological activities in the systemic circulation, such as cytoprotective, antioxidative, anti-inflammatory, and vasoprotective properties.^14^ Herein, we demonstrate that ARC treatment significantly prevented pulmonary artery remodeling, the reversed mean pressure of pulmonary artery elevation, and RV hypertrophy in MCT-treated rats. Moreover, our results suggest that ARC may attenuate the progression of MCT-induced PAH by reducing the expression of inflammatory cytokines, inhibiting the proliferation of pulmonary arterial smooth muscle cells, decreasing oxidative stress, and downregulating the NLRP3 signal pathway in lungs.

In agreement with human pulmonary hypertension, marked medial wall thickening was observed in MCT-induced pulmonary hypertension rats. We found a marked reduction in the progression of pulmonary hypertension and medial wall thickening in response to ARC treatment (Fig. 1A–C). Accordingly, adaptive hypertrophy in the right ventricle of MCT-treated rats was also prevented by ARC (Fig. 1D). Therefore, the present results indicate that ARC treatment prevents pulmonary arterial remodeling and exerts a cardiopulmonary protective effect, at least in the MCT-induced PAH rat model.

The aberrant proliferation of PASMCs is a pathological hallmark of PAH.^15^ The present study shows that smooth muscle cell proliferation was markedly increased in pulmonary resistance vessels of MCT-treated animals. ARC treatment induced near normal vessel morphology and inhibited pulmonary arterial smooth muscle proliferation (Fig. 2A–C). Our *in vitro* study demonstrated that ARC prevented PDGF-induced proliferation in cultured PASMCs without increasing apoptotic cell death in PASMCs (Fig. 3). Therefore, it is likely that the inhibition of pulmonary arterial smooth muscle proliferation and vascular remodeling is predominantly attributed to the direct effect of ARC.

Increasing evidence supports an important role for inflammation in the development and progression of human pulmonary hypertension and experimental animal models.^16^ For example, in various forms of clinical pulmonary hypertension, there is increasing recognition of inflammation on the basis of evidence including increased plasma levels of inflammatory cytokines and the pulmonary infiltration of inflammatory cells.^17^ Similarly, in animal models of pulmonary hypertension, increased numbers of macrophages have been described in mouse and rat lungs.^17^ These processes might contribute to vascular remodeling, collagen deposition, and PASMC proliferation and migration in PAH, and eventually lead to pulmonary resistance and subsequent right heart failure. IL-1β is a prototypic multifunctional cytokine that has a vital role in the pathogenesis of PAH.^18,19^ In agreement with previous findings, IL-1β was increased in MCT-treated rats, and other proinflammatory cytokines were also upregulated (Fig. 4). In addition, ARC, a natural anti-inflammatory agent, has shown apparent protective effects in LPS-induced acute lung injury mouse model at a dose of 50 mg per kg per day.^9^ Here, we found that ARC treatment in MCT-treated rats normalized the expression of inflammatory cytokines (Fig. 4).

In addition to inflammatory reactions, incremental evidence suggests that oxidative stress may impair the pulmonary vascular endothelium and induce the proliferation of vascular muscle cells in rat models of PAH.^3^ Moreover, increased oxidative stress was observed in PAH patients, while the reduction of oxidative stress was associated with an attenuation of the clinical symptoms.^20^ It was previously reported that ARC reduced oxidative stress in many diseases, in line with our finding that treatment with ARC significantly downregulated MDA levels and increased SOD activity (Fig. 5A and B). Therefore, the antioxidative effects of ARC may also contribute to blocking the progression of MCT-induced PAH in rats.

The mechanisms by which the antioxidative effects of ARC interfere with inflammatory processes in the lung are not well understood. Recently, several studies have highlighted the role of NLRP3 inflammasome in several types of lung injury. NLRP3-deficient mice showed a suppressed inflammatory response and blunted lung epithelial cell apoptosis in hyperoxia-induced acute lung injury.^21^ In addition, ROS is central to NLRP3 activation, which is critical for the release of caspase-1 and IL-1β.^6,22^ Of note, the roles of inflammation, caspase-1, and IL-1β in the pathogenesis of experimental PAH have been reported by many studies.^23^ Recently it was shown that ARC effectively blocked NRLP3 activation in neurons, thus protecting oxygen glucose deprived neurons.^10^ In accordance with previous studies, our present study implies that ARC inhibits the NLPR3 inflammasome in MCT-treated rats. On the basis of the respective roles of oxidative stress, inflammation, and IL-β in the pathogenesis of PAH, as well as the interactions between reactive oxidative species, the inflammasome, and IL-1β, we propose that the antioxidative effect of ARC on PAH may contribute to the inhibition of the NLRP3 inflammasome *via* oxidative stress inhibition.

In conclusion, we demonstrated that ARC treatment prevented the development of MCT-induced PAH by significantly attenuating pulmonary vascular remodeling, increasing RVSP, RV hypertrophy, and cardiomyocyte enlargement. These beneficial effects of ARC might be attributed to antiproliferative, anti-inflammatory, and antioxidative actions, which are mediated, at least in part, *via* inhibition of the NLRP3 inflammasome signaling pathway. Future studies are needed to confirm these specific mechanisms and to determine whether ARC treatment can reverse or delay established PAH.

## Conflicts of interest

None.

## Supplementary Material
